# Governing the pandemics: Moving towards an assertive institutional environment

**DOI:** 10.7189/jogh.11.03021

**Published:** 2021-01-30

**Authors:** Min Jung Cho, VS Saravanan, Eun Jung Kim

**Affiliations:** 1 Global Public Health, Leiden University College, Faculty of Global Governance and Affairs, The Hague, the Netherlands; 2 Department of Civil, Geo and Environmental Engineering, Technical University Munich, Garching, Germany; 3 School of Architecture, Hanyang University, Seoul, South Korea

The global pandemic of coronavirus should be a clarion call to revamp global and national health institutions and their approaches. This new virus exhibits high transmissibility and with no medical countermeasures poses a risk to health systems worldwide. A few months prior to this pandemic, the Global Preparedness Monitoring Board (GPMB) warned that ‘there is a very real threat of a rapidly moving, highly lethal pandemic of a respiratory pathogen killing 50 to 80 million people and affecting nearly 5% of the world's economy [1]. Unfortunately, the world did not expect that this would strike soon.

Health risk is interconnected with social well-being, economic forces, and human rights. This requires a fundamental shift in understanding the complex nature of health to build solid, cost-effective preventive actions and policies in the future. Unfortunately, pandemics and epidemics are largely understood and dealt with from a biomedical approach. The COVID-19 pandemic is a reminder to move beyond this reductionist perspective towards an assertive institutional environment, especially when there is inadequate scientific understanding and uncertainty prevailing in the event. An assertive institutional environment is about the actors taking a step back from their predetermined concepts and frameworks to take decisions as an external viewer for the welfare and security of the humanity and their ecology nationally and globally. This requires stronger international cooperation, adequate foresights, social solidarity, and optimized resources with strong leadership and effective communication.

## INTERNATIONAL COOPERATION

Governance during a pandemic is about setting formal and informal rules, mobilizing public institutions, enacting regulations, reinforcing socially embedded norms and values, and using market forces to integrate within and beyond nations to manage the crisis. There are two international frameworks that guides national and international cooperation during health emergencies. The first is the International Health Regulations (IHR), which remain at the core for international cooperation with over 196 national signatories, which came into force in 2007 and was updated in 2009 [2]. Yet, after more than 10 years, the IHR and its revised version have proven to be inadequate and have cast doubts on the effectiveness of the regulations. The second is the WHO Guidelines [3] for influenza preparedness and response, which identifies five basic components: (i) planning and coordination, (ii) situation monitoring and assessment, (iii) reducing the spread of disease, (iv) continuity of health care provision, and (v) communications. Interestingly, during the COVID-19 pandemic these frameworks only offered guidelines for countries to share information on the characteristics of the virus to the international community.

There were a few regional and bilateral initiatives during this pandemic, which offers significant lessons for cooperation and sharing during the pandemics. The African Union (AU) played an effective role in communicating about and shaping African responses, with technical legitimacy provided through the Africa Centres for Disease Control and Prevention (Africa CDC) [4]. The regional responses within AU reflected a spectrum of cooperation, complexity, and the politics of diplomacy during pandemics – rising from information sharing; to ‘nudging’ and guiding; to active coordination of state responses, to collective action. Bilaterally, many countries were engaged in the dispatch of personal protective equipment’s. China provided pandemic-related aid to personal protective equipment, including 1000 for the New York city. Germany offered to provide hospital beds to treat critically ill patients from neighbouring countries demonstrating the European solidarity [5]. Despite these initiatives, international institutions have an immense task to address the shared global problems that are likely to linger, such as movement restrictions, high levels of unemployment, rising dissatisfaction with governing institutions and growing civil liberties during pandemics. This requires international institutions to strengthen international cooperation through adequate foresight, building social solidarity, and enlightened leadership [6,7].

## FORESIGHT

Foresight remains crucial for national- and international-level preparedness and response during pandemics when there is uncertainty in the behaviour and unpredictably in the scientific understanding of the pathogens. China realized the seriousness of the virus to activate the Epidemic Prevention and Control Headquarters System (EPCHS), set up the Joint Prevention and Control Mechanism of the State Council (JCMSC) and mobilized heath work force [8], many of these measures were non-pharmaceutical interventions. This was supported by transforming public venues to hospitals and make-shift hospitals to ensure health security. In late January, when South Korea came to know about the virus outbreak in China, the country’s health officials, and representatives from more than 20 medical companies met to discuss the manufacture of testing kits and the details of government support [9]. Their rapid response to COVID-19 was partially a result of their previous experience with SARS 2003 and MERS. India, realizing its inadequacies in health infrastructure in a highly dense and unequal society, invoked the National Disaster Management Act-2005 to impose nation-wide lockdown as a non-pharmaceutical intervention to contain the initial spread of virus [10]. When cases were reported in China, Germany developed the diagnostic test kits for COVID-19 and mobilized the country`s public and private laboratories to rapidly scale up testing capacity [11]. This was followed by its ability to manage the infection rates in hospital and long-term care facilities [12]. It is this leadership during a pandemic with public awareness of the prevailing uncertainty and bold decisions by considering the interests of public, private, and civil society that played a significant role in the initial containment of the disease spread.

**Figure:**
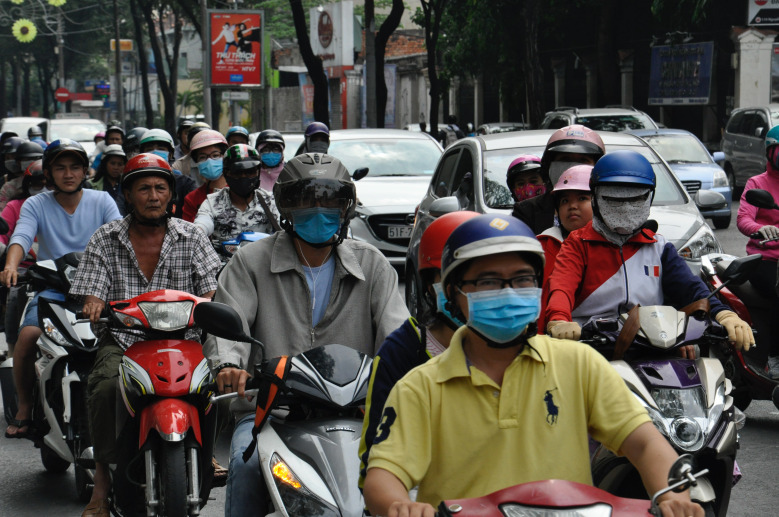
Photo: From Unsplash (https://unsplash.com/photos/8BIy9ifXMtA).

## SOCIAL SOLIDARITY

Motivating stakeholders to pay attention to foresight and assertive action requires more than just sophisticated expertise in communication and building solidarity. Diverse social organizations in China participated in the epidemic control through shared expertise, promoting public health literacy, and through volunteering programs. This helped to disseminate information and gain social support [8]. Solidarity can also be aided by experiential backing. Having learnt lessons from the MERS and SARS epidemics, people in South Korea gave a solid backing to their government. Similarly, when China announced the pandemic, good social communication from the government and sub-national agencies with the public played a significant role in containing and controlling pandemic. The Indian prime minister took primary responsibility in reaching out to the common public (where most of them are illiterate) through the weekly address on television to highlight the seriousness of the pandemic and sort support from the citizens while imposing the countrywide lockdown [10]. The Robert Koch Institute in Germany took lead in publishing risk assessment strategy document, response plans, daily surveillance reports and technical guidelines which formed the basis for public awareness and critical decisions by stakeholders during the outbreak [12]. It can be a challenge for countries to mobilize the public for massive state intervention, and this is often only possible in a crisis like COVID-19. From the outset, decision-making in these countries has been a collaboration between the government and the scientific community, solidarity from their citizens and a highly modern testing system.

## OPTIMIZING TECHNOLOGY

Technology and in-house resources have been useful for many nations in containing the spread of the pandemic. For example, the Smart Management System developed by South Korea to track and analyse the movement of infected individuals [13] was useful to that country in containing COVID-19. The technology gave epidemiological investigators real-time data about the patients, their contacts, and their movements to enable tracking infection routes for effective containment and treatment. Countries that have maintained low COVID-19 per capita mortality rates appear to share strategies that relied on digital technology and integrating it into policy and health care [14]. The future of public health is likely to become increasingly digital, and to succeed we need for the alignment of international strategies for the regulation, evaluation and use of digital technologies to strengthen pandemic management, and future preparedness for COVID-19 and other infectious diseases [15].

## CONCLUSION

The world has not experienced a simultaneous and indiscriminate social, health, governance, and economic crisis to rival that experienced because of COVID-19 [7]. This has exposed not only how far the world is from effective and unified global governance, but also multiple crises of confidence in the institutions expected to guide international action and cooperation [7]. The Viewpoint calls for revision of the international guidelines and national approach towards facilitating an assertive institutional environment for pandemic governance. An assertive institutional environment is about the actors stepping back to take decisions as an external viewer for the welfare and security of humanity and their ecology nationally and globally. It is not about having a proactive policy with predictable concrete events but rather drawing attention to potentially relevant developments on an everyday basis recognizing the prevailing uncertainty, unpredictability, and availability of resources with strong leadership [16]. It is about addressing them through the interplay of consolidated interests, political competitiveness, and more urgent matters on the everyday agenda. This requires stronger international cooperation, adequate foresights, stronger solidarity among citizens and governments, and optimized resources with strong leadership and effective communication. It is important that such a framework goes even further by integrating with diverse health care systems; incorporating economic and market behaviour; regulating social behaviour and resource transfer in their day-to-day movements, addressing socio-economic effects, developing socially informed and acceptable measures, and ensuring equitable access to health security through national and international cooperation. The pandemic also presents an opportunity for country agencies to improve education regarding hygiene and other practices, strengthen their health infrastructure, improve immunity-based diet systems, improve housing, and enhance cooperation among nations and intergovernmental agencies to help develop governance structures in alignment with other health-related sectors.
